# Chlorogenic acid protects against cisplatin-induced testicular damage: a biochemical and histological study

**DOI:** 10.2478/aiht-2025-76-3990

**Published:** 2025-06-30

**Authors:** Elif Ayazoğlu Demir, Selim Demir, Sevdegül Aydın Mungan, Nihal Türkmen Alemdar, Ahmet Menteşe, Yüksel Aliyazıcıoğlu

**Affiliations:** Karadeniz Technical University Macka Vocational School, Department of Chemistry and Chemical Processing Technologies, Trabzon, Turkey; Karadeniz Technical University Faculty of Health Sciences, Department of Nutrition and Dietetics, Trabzon, Turkey; Karadeniz Technical University Faculty of Medicine, Department of Medical Pathology, Trabzon, Turkey; Recep Tayyip Erdogan University Vocational School of Health Services, Department of Medical Services and Techniques, Rize, Turkey; Karadeniz Technical University Faculty of Medicine, Department of Medical Biochemistry, Trabzon, Turkey

**Keywords:** apoptosis, endoplasmic reticulum stress, HO-1, inflammation, Nrf2, oxidative stress, apoptoza, HO-1, Nrf2, oksidacijski stres, stres endoplazmatskoga retikuluma, upala

## Abstract

One of the adverse effects of cisplatin (CIS) treatment is its reproductive toxicity, which limits its clinical use in male patients. The aim of our study was to investigate the potential protective effects and mechanisms of chlorogenic acid (CHA), a well-known antioxidant and anti-inflammatory polyphenol, in a CIS-induced testicular toxicity model. To this end we divided 30 Sprague-Dawley rats into five groups: control and four groups receiving either CHA alone (3 mg/kg), CIS alone (5 mg/kg), or their weaker and stronger combinations: CIS+CHA (1.5 mg/kg) and CIS+CHA (3 mg/kg), respectively. In the combination groups the rats first received a single 5 mg/kg dose of CIS, followed by either 1.5 or 3 mg/kg of CHA administered intraperitoneally for three consecutive days. Testicular tissues were harvested on the fifth day of the experiment. The level of testicular oxidative stress and inflammation induced by CIS and the histopathological changes observed were restored to normal following treatment with both doses of CHA. Furthermore, treatment with CHA led to the regeneration of Nrf2 and HO-1 levels, which had been suppressed by CIS. Consequently, the levels of endoplasmic reticulum stress and apoptosis were reduced. These findings indicate that CHA may counter the reproductive toxicity of CIS and may therefore serve as its add-on in cancer therapy.

## Abstract (hr)

Jedan od štetnih učinaka cisplatina je njegova reproduktivna toksičnost, koja ograničava kliničku primjenu ovog antitumorskog lijeka u muškaraca. Cilj našeg istraživanja bio je ispitati moguće zaštitne učinke i mehanizme djelovanja klorogenske kiseline, poznatog antioksidansa i protuupalnog polifenola, na modelu toksičnosti za testise prouzročene cisplatinom. U tu smo svrhu podijelili 30 štakora soja Sprague-Dawley u pet skupina: kontrolnu skupinu i četiri skupine koje su primale samo klorogensku kiselinu (3 mg/kg), samo cisplatin (5 mg/kg) ili njihovu kombinaciju s nižom ili višom dozom klorogenske kiseline (1,5 odnosno 3 mg/kg). U kombiniranim skupinama štakori su prvo primili jednokratnu dozu cisplatina (5 mg/kg), a zatim su tri uzastopna dana intraperitonealno primali klorogensku kiselinu u nižoj odnosno višoj dozi. Tkivo testisa prikupljeno je petog dana pokusa. Razina oksidacijskoga stresa i upale u testisima izazvane cisplatinom te promjene u histološkoj građi vraćene su na normalne vrijednosti nakon primjene obiju doza klorogenske kiseline. Nadalje, primjena klorogenske kiseline dovela je do obnove razina Nrf2 i HO-1 nakon prvotne inhibicije cisplatinom. Posljedično su se smanjile razine stresa endoplazmatskoga retikuluma i apoptoze. Ovi rezultati upućuju na to da klorogenska kiselina može ublažiti reproduktivnu toksičnost cisplatina te stoga poslužiti kao dodatak u liječenju raka.

Cisplatin (CIS) is one of the most common agents in the treatment of lung, ovarian, and head and neck tumours (1). Its antitumoral activity is owed to its potential to form intra- and inter-crosslinks in the DNA helical structure (2). Such formation of DNA adducts in cancer cells disrupts the nuclear function, which in turn causes cell cycle arrest and apoptosis (3). Furthermore, recent studies have demonstrated that CIS-induced production of reactive oxygen species (ROS) contributes to its antitumoral activity (4, 5). However, like other chemotherapeutics, CIS can damage normal cells and be nephro-, oto-, hepato-, reno- and reprotoxic (1, 3). Experimental studies have demonstrated that CIS exerts its detrimental effects on male reproductive organs by suppressing the antioxidant system, which is comprised of superoxide dismutase (SOD), glutathione peroxidase (GPx), and glutathione (GSH). This process results in lipid peroxidation (LPO) and increased levels of antigenic intermediates such as malondialdehyde (MDA) (1, 5). CIS-induced oxidative stress (OS) also accelerates inflammatory cell damage by activating the nuclear factor kappa-B (NF-κB) pathway and by increasing the levels of pro-inflammatory cytokines, including interleukin-6 (IL-6) (3). Furthermore, CIS can reduce circulating testosterone levels by suppressing the expression of the steroid acute regulatory protein, which in turn leads to changes in testicular morphology characterised by impaired spermatogenesis (1). This may limit clinical treatment options and result in chronic subfertility and infertility (6).

Some studies have therefore proposed that antioxidant molecules may be beneficial in preventing CIS-induced tissue toxicity (5, 7). Considering, however, that synthetic antioxidants and anti-inflammatory molecules can damage healthy tissue in chronic use, research has shifted interest to secondary metabolites originating from natural products (8). One such natural product is chlorogenic acid (CHA), a polyphenol found in coffee, carrot, kiwi, tea, and pear (9). It has the potential to modulate the nuclear factor-erythroid 2-related factor 2 (Nrf2) pathway (10,11,12), which controls the expression of antioxidative enzymes, including SOD, GPx, and haem oxygenase 1 (HO-1) involved in maintaining redox homeostasis in cells (13). As this pathway has been reported to be inhibited by CIS (14,15,16), one way to eliminate chemotherapy-induced healthy tissue toxicity is to restore it. However, a cautious approach is warranted, because Nrf2 may inadvertently improve the survival of cancer cells in an organism and therefore increase resistance to chemotherapy (13). Although the beneficial effects of CHA against testicular damage caused by chemicals such as tamoxifen, methotrexate, and tunicamycin have been reported earlier (17,18,19), no study has yet demonstrated the effects of CHA against CIS-induced male reprotoxicity. The aim of our study was to address this gap by investigating the protective effects of CHA against CIS in testicular tissue and to evaluate its therapeutic potential.

## MATERIALS AND METHODS

### Animals

A total of 30 male Sprague-Dawley rats (200–220 g) were obtained from the Karadeniz Technical University Surgery Application and Research Centre. The animals were housed there under standard conditions (22±1 °C and a 12 h dark/light cycle) with free access to food and water. This study was approved by the Karadeniz Technical University Ethics Committee for Experimental Animals (approval no. 2022/46), and all experiments were performed with a humane approach in accordance with the ARRIVE guidelines (20) and EU Directive 2010/63/EU (21).

### Experimental procedure

After one-week of adaptation, the rats were divided into five groups of six, all of which were receiving intraperitoneal injections. The control group received a saline on the 1^st^ day and 10 % dimethyl sulphoxide (DMSO) for the following three days. The CHA group received saline on the 1^st^ day and 3 mg/kg of CHA (dissolved in 10 % DMSO) for the following three days. The CIS group received a single 5 mg/kg dose of CIS (dissolved in saline) on the 1^st^ day and 10 % DMSO for the following three days. The CIS + low-dose CHA group received CIS as described above on the 1^st^ day and CHA (1.5 mg/kg) injection for the following three days. The CIS + high-dose CHA group received CIS as described above on the 1^st^ day, followed by CHA (3 mg/kg) for the following three days.

The CIS dose used in this study was based on previous reports of its testicular toxicity in experimental studies (22, 23). The choice of the relatively low CHA doses (1.5 or 3 mg/kg) was based on the wish to avoid adverse effects reported elsewhere (24, 25) and on previous reports confirming their effectiveness (26,27,28). The five-day duration of the experiment was based on reports indicating that the intraperitoneal application of herbal phytochemicals elicits therapeutic effects in models of CIS-induced acute toxicity within this time frame (22, 23, 29, 30).

### Sample collection

Four days after the initial injection, all animals were euthanised by exsanguination under general anaesthesia with ketamine (60 mg/kg) and xylazine (10 mg/kg). Testicles were removed immediately and one half stored at −80 °C, while the other half was fixed in Bouin's solution.

### Tissue preparation

Testicular tissues (approximately 30 mg) were homogenised in phosphate-buffered saline (PBS) (pH 7.4), centrifuged at 1800×*g* and 4 °C for 10 min, and protein levels in the resulting supernatants determined with a commercial Pierce BCA Protein Assay Kit (Thermo Scientific, Rockford, IL, USA) using bicinchoninic acid (BCA) as described by Smith et al. (31). Briefly, 25 μL of serially diluted bovine serum albumin (BSA) standards and supernatants (diluted ten-fold with PBS) were dispensed into a 96-well microplate, 200 μL of BCA working reagent was added to each well and left to incubate at 37 °C for 30 min. The absorbance of the standards and supernatants was read on a spectrophotometer (VersaMax microplate reader, Molecular Devices, Sunnyvale, CA, USA) at 562 nm and plotted against concentration graph for the BSA standards to measure protein content in the samples.

### Determination of lipid peroxidation

The LPO level in supernatants was determined by quantifying MDA levels as described elsewhere (32). The standard used was 1,1,3,3-tetramethoxypropane. The absorbances of the samples and standards were measured at a wavelength of 532 nm, and tissue MDA levels are expressed in nmol/mg of protein.

### Determination of antioxidative capacity

The levels of antioxidative enzymes and Nrf2 were determined with commercial ELISA kits provided by Bostonchem (Boston, MA, USA) [SOD (Cat No.: BLS-8178Ra), GPx (Cat No.: BLS-2222Ra), and GSH (Cat No.: BLS-8577Ra)] or by Finetest (Wuhan, China) [Nrf2 (Cat No.: ER0666) and HO-1 (Cat No.: ER1041)] and are expressed in ng, pg, μg, pg, and ng per mg of protein, respectively. Briefly, the primary stock standard provided by the manufacturers was subjected to serial dilution. Subsequently, 100 μL of both samples and standards were added to each well of an antibody-coated plate, after which the plate was incubated on a shaker at 37 °C for 90 min. The wells were then washed, added 100 μL of biotin-labelled antibody solution, and incubated for another 60 min. Followed the second washing step, adding 100 μL of streptavidin-HRP solution, and incubation for another 30 min. After the third washing step, we added 90 μL of TMB substrate solution to each well, and repeated the incubation for another 20 min. Finally we added 50 μL of stop solution to each well to halt the reaction. The absorbances of the samples and standards were read on the VersaMax plate reader (Molecular Devices) at a wavelength of 450 nm and their levels calculated as described elsewhere (22).

### Determination of inflammation parameters

To determine inflammation parameters we used the Finetest ELISA kit for NF-κB p65 (Cat No.: ER1187) or the Bostonchem kit for IL-6 (Cat No.: BLS-1158Ra) and myeloperoxidase (MPO) (Cat No.: BLS-1661Ra). The levels of NF-κB p65, IL-6, and MPO are expressed in pg, pg, and ng per mg of protein, respectively.

### Determination of endoplasmic reticulum stress and apoptosis

Commercial ELISA kits (Bostonchem, Boston, MA, USA) were also used to measure the levels of the heat shock protein family A member 5 (HSPA5) (Cat No.: BLS-6834Ra), activating transcription factor 6 (ATF6) (Cat No.: BLS-9545Ra), DNA damage-inducible transcript 3 (DDIT3) (Cat No.: BLS-8868Ra), and cleaved caspase-3 (CASP3) (Cat No.: BLS-1528Ra). The levels of all ERS and apoptosis markers are expressed in ng per mg of protein.

### Histological analysis

Testicular tissues fixated in Bouin's solution for 48 h were analysed as described elsewhere (23, 33). Briefly, 5 μm-thick sections were cut from the prepared paraffin blocks with a microtome (Leica RM2255, Wetzlar, Germany), stained with haematoxylin-eosin (H&E), analysed by a blinded pathologist under a light high-powered microscope (Olympus BX51, Tokyo, Japan) with ×200 magnification, and photographed. Maturation of the germinal epithelium was assessed using a modified Johnsen testicular biopsy score (34, 35). A total of 20 tubules were assessed for each preparation. Each tubule was scored in the range from 1 to 10, where 1 indicates complete absence of germ cells and 10 maximum spermatogenic activity.

### Statistical analysis

The required sample size of six animals per group was determined using the G*Power v 3.1.9.2 statistical software (University of Kiel, Kiel, Germany) to ensure adequate power to detect potential significant differences in parameters (1–β=0.8), specified effect size of 2.0, implementation of a two-sided *t*-test, and the sample size ratio of 1.

The obtained data were analysed using the SPSS 23.0 software (IBM, Chicago, IL, USA). All values are expressed as group means ± standard errors of the mean (SEM). The normality of the data distribution was established with the Shapiro-Wilk test. One-way ANOVA and post-hoc Tukey's test were employed to compare normally distributed data and histological scores across all experimental groups. The p value of <0.05 was considered statistically significant.

## RESULTS

### Oxidative stress parameters

**Figure 1:**
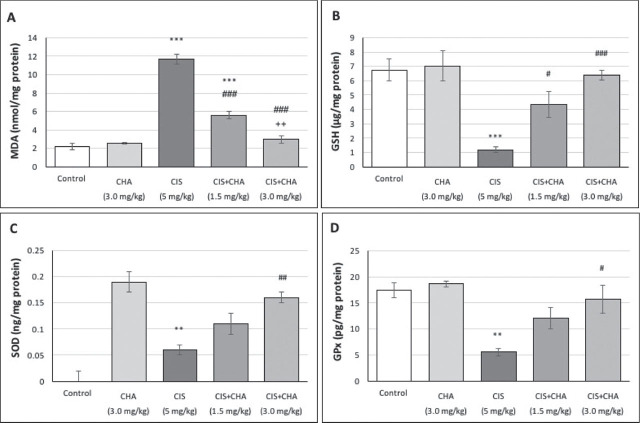
Effects of CHA (1.5 and 3 mg/kg) on OS parameters in a CIS-induced testicular toxicity rat model. Data are presented as means ± SEM. ^**^p<0.01 and ^***^p<0.001 – significant difference from control; ^#^p<0.05, ^##^p<0.01, and ^###^p<0.001 – significant difference from the CIS alone group; ^++^p<0.01 – significant difference from the CIS+CHA (1.5 mg/kg) group. CHA – chlorogenic acid; CIS – cisplatin; GPx – glutathione peroxidase; GSH – reduced glutathione; MDA – malondialdehyde; OS – oxidative stress; SEM – standard error of the mean; SOD – superoxide dismutase

Figure 1 shows changes in MDA, SOD, GPx, and GSH levels in rat testicular tissue. Treatment with CIS resulted in a significant increase in MDA levels (~5.3-fold) and a significant decrease in GSH (~5.7-fold), SOD (~2.5-fold), and GPx (~3.1-fold) levels compared to control. The three-day treatment with CHA following CIS countered these effects in a dose-dependent manner by lowering MDA and increasing antioxidative parameters. The administration of the higher CHA dose alone did not result in any adverse effects.

### Inflammation parameters

**Figure 2:**
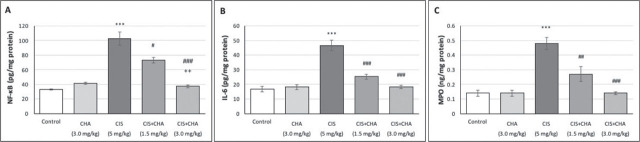
Effects of CHA (1.5 and 3 mg/kg) on inflammation parameters in a CIS-induced testicular toxicity rat model. Data are presented as means ± SEM. ^***^p<0.001 – significant difference from control; ^#^p<0.05, ^##^p<0.01 and ^###^p<0.001 – significant difference from the CIS alone group; ^++^p<0.01 – significant difference from the CIS+CHA (1.5 mg/kg) group. CHA – chlorogenic acid; CIS – cisplatin; IL-6 – interleukin-6; MPO – myeloperoxidase; NF-κB – nuclear factor kappa B; SEM – standard error of the mean

Figure 2 shows a significant rise in testicular NF-κB p65 (~3.1-fold), IL-6 (~2.8-fold), and MPO (~3.4-fold) levels in rats administered with CIS alone compared to control. The three-day administration of CHA countered these effects in a dose-dependent manner. Again, the higher CHA dose alone did not have any adverse effects.

### ERS and apoptosis

**Figure 3:**
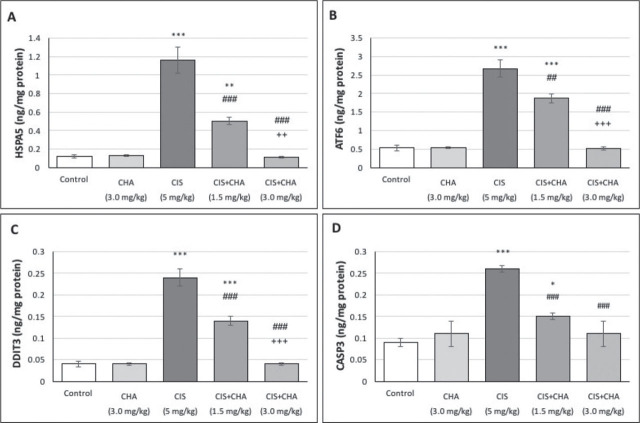
Effects of CHA (1.5 and 3 mg/kg) on ERS and apoptosis parameters in a CIS-induced testicular toxicity rat model. Data are presented as means ± SEM. ^*^p<0.05, ^**^p<0.01 and ^***^p<0.001 – significant difference from control; ^##^p<0.01 and ^###^p<0.001 – significant difference from the CIS alone group; ^++^p<0.01 and ^+++^p<0.001 – significant difference from the CIS+CHA (1.5 mg/kg) g roup. ATF6 – activating transcription factor 6; CASP3 – cleaved caspase-3; CHA – chlorogenic acid; CIS – cisplatin; DDIT3 – DNA damage-inducible transcript 3; ERS – endoplasmic reticulum stress; HSPA5 – heat shock protein family A (HSP70) member 5; SEM – standard error of the mean

Figure 3 shows that the single CIS dose significantly increased HSPA5 (~9.7-fold), ATF6 (~5.0-fold), DDIT3 (~6.0-fold), and CASP3 (~2.9-fold) levels compared to control. As with other parameters, the three-day CHA administration significantly lowered these levels in a dose-dependent manner. Its higher dose alone did not result in any adverse effects.

### Nrf2/HO-1 pathway

**Figure 4:**
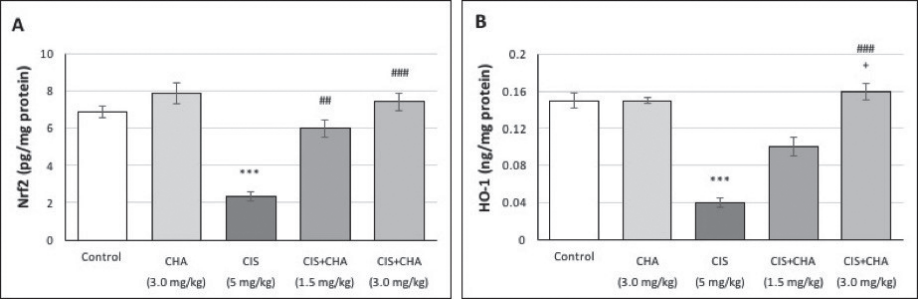
Effects of CHA (1.5 and 3 mg/kg) on the Nrf2/HO-1 pathway in a CIS-induced testicular toxicity rat model. Data are presented as means ± SEM. ^***^p<0.001 – significant difference from control; ^##^p<0.01 and ^###^p<0.001 – significant difference from the CIS alone group; ^+^p<0.05 – significant difference from the CIS+CHA (1.5 mg/kg) group. CHA – chlorogenic acid; CIS – cisplatin; HO-1 – haem oxygenase-1; Nrf2 – nuclear factor erythroid 2-related factor 2; SEM – standard error of the mean

Figure 4 shows that CIS administration resulted in a ~3.0-fold suppression of Nrf2 and ~3.8-fold suppression of HO-1 compared to control. CHA treatment restored them in a dose-dependent manner and did not adversely affect these parameters in the CHA group.

### Histopathological changes in testicular tissue

**Figure 5:**
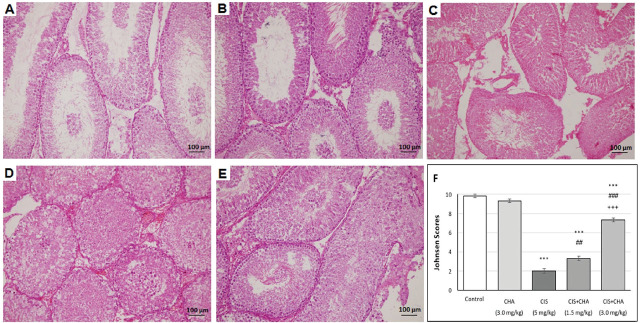
Effects of CHA (1.5 and 3 mg/kg) on testicular tissue architecture in CIS-treated rats (200×). A) control; B) CHA alone (3 mg/kg); C) CIS (5 mg/kg); D) CIS+CHA (1.5 mg/kg); E) CIS+CHA (3 mg/kg); F) Mean Johnsen scores for all groups. Data are presented as means ± SEM. ^***^p<0.001 – significant difference from control; ^##^p<0.01 and ^###^p<0.001 – significant difference from the CIS alone group; ^+++^p<0.001 – significant difference from the CIS+CHA (1.5 mg/kg) group. CHA – chlorogenic acid; CIS – cisplatin; SEM – standard error of the mean

Figure 5 shows typical histopathological changes and semiquantitative scores on the Johnsen scale across all groups. While numerous seminiferous tubule structures with normal spermatogenic activity were observed in both the control and high-dose CHA group, CIS caused severe necrosis of the seminiferous tubules, as evidenced by the markedly lower Johnsen scores. Treatment with low-dose CHA partly improved the spermatogenic activity, characterised by a small number of spermatogonia. Treatment with high-dose CHA resulted in a significant improvement in pathological findings, as evidenced by higher Johnsen scores.

## DISCUSSION

Our findings confirm earlier reports of CIS toxicity disrupting testicular architecture (23, 36, 37) through oxidative stress (23, 36, 38, 39) as the primary mechanism, which interrupts various signalling pathways, Nrf2 in particular (14,15,16), and triggers inflammation (14, 36, 40, 41) and ERS-induced apoptosis (42,43,44,45) to eventually lead to tissue damage (3, 5).

More importantly, CHA delivered on promising findings reported earlier in all aspects of our experiment. In a dose-dependent manner it nearly restored redox homeostasis, most likely by scavenging free radicals and activating the antioxidant signalling pathway, which is in line with antioxidant activity reported earlier (25, 46,47,48). It reactivated the Nrf2 pathway (36), most likely inhibited by ERS (44) and consequently reduced inflammatory response to CIS (49) and apoptosis, which is consistent with previous reports of ERS inhibition and anti-apoptotic (19, 50, 51) as well as the anti-inflammatory activity of CHA (52,53,54). Eventually, this led to marked improvements in testicular tissue findings evidenced by higher Johnsen scores. These findings are consistent with previous reports indicating the testicular protective effects of CHA (50, 55).

In conclusion, our study provides initial evidence of the beneficial effects of CHA against CIS-induced reproductive toxicity. These results require further corroboration through comprehensive molecular and physiological investigations before clinical implementation as an add-on in cancer therapy with CIS.

## List of abbreviations

ATF6: activating transcription factor 6
CASP3: cleaved caspase-3
CHA: chlorogenic acid
CIS: cisplatin
DDIT3: DNA damage-inducible transcript 3
DMSO: dimethyl sulfoxide
ERS: endoplasmic reticulum stress
GPx: glutathione peroxidase
GSH: glutathione
H&E: haematoxylin and eosin
HO-1: haem oxygenase-1
HRP: horseradish peroxidase
HSPA5: heat shock protein family A (HSP70) member 5
IL-6: interleukin-6
Keap1: Kelch-ECH-associated protein 1
LPO: lipid peroxidation
MDA: malondialdehyde
MPO: myeloperoxidase
NF-κB: nuclear factor kappa B
Nrf2: nuclear factor erythroid 2-related factor 2
OS: oxidative stress
ROS: reactive oxygen species
SEM: standard error of the mean
SOD: superoxide dismutase
TMB: 3,3′,5,5′-tetramethylbenzidine
